# The Exempts

**Published:** 1891-10

**Authors:** 


### THE EXEMPTS.

THE *Post Graduate*, one of the most enterprising quarterly journals, is doing splendid work in the cause of higher medical education. Its editorial columns for July fairly bristle with pointed paragraphs and sayings, from which we regret we have only space to extract the following:

One of the most absurd claims of the gentlemen who succeeded in passing this bill exempting certain students from the Medical Examiners' Law, was that there was a contract made with the medical students of the Long Island College Hospital, by which it was promised that they would be graduated under the old law. This is all nonsense. There can be no contract unless there are two parties. Did any one of these students agree to stay under the then existing law, until he received his diploma? Not one. Medical students never make any such contract. They attend one course at one college, another at another, and so on. But even if they did, every college in this State knew that at any moment the Legislature was likely to change the existing conditions. They began the study of medicine with this knowledge. The college could not have made such a contract, even if the students had asked them to, and that they did was an after-thought. No injustice was done these gentlemen. They ought to have been glad that they were to be the first to inaugurate the new law in this State, but they have chosen the ignominious position of begging to be released from bearing an obligation they ought to have been proud to assume. They will live long enough, we trust, to regret having put anything in the way of elevating the scientific and social position of the profession into which they are to enter.

